# Adaptations for TAP in a Long-Term Care Setting

**DOI:** 10.1093/geroni/igaa057.2158

**Published:** 2020-12-16

**Authors:** Katherine Marx, Lauren Parker, Laura Gitlin

**Affiliations:** 1 Johns Hopkins University, Baltimore, Maryland, United States; 2 Johns Hopkins Medicine, Baltimore, Maryland, United States; 3 Drexel University, Philadelphia, Pennsylvania, United States

## Abstract

One of the most difficult aspects of caring for people living with dementia is managing neuropsychologic symptoms and functional decline. Although there are hundreds of efficacious non-pharmacologic interventions tested in homes, few are adapted for and tested in long-term care. The purpose of this pilot study was to identify the adaptations needed for the Tailored Activity Program (TAP) to make it feasible and acceptable in a long-term care facility. TAP provides tailored activities matched to interests and abilities to address dementia-related clinical symptoms. Two sites, under the umbrella of one company, participated. A total of five persons living with dementia, their family caregivers, two direct care staff and an interventionist participated, and occupational therapist who contracts with the site on a regular basis. Adaptations included shorter sessions and changes to forms to fit with workflows and documentation. Additional considerations challenging implementation of TAP included staff turn-over and training. Part of a symposium sponsored by the Behavioral Interventions for Older Adults Interest Group.

